# Plant Secondary Metabolite-Derived Polymers: A Potential Approach to Develop Antimicrobial Films

**DOI:** 10.3390/polym10050515

**Published:** 2018-05-10

**Authors:** Ahmed Al-Jumaili, Avishek Kumar, Kateryna Bazaka, Mohan V. Jacob

**Affiliations:** 1 Electronics Materials Lab, College of Science and Engineering, James Cook University, Townsville, QLD 4811, Australia; Ahmed.Aljumaili@my.jcu.edu.au (A.A.-J.); Avishek.kumar@my.jcu.edu.au (A.K.); kateryna.bazaka@qut.edu.au (K.B.); 2 Physics Department, College of Science, Ramadi, Anbar University, Ramadi 11, Iraq; 3 School of Chemistry, Physics, Mechanical Engineering, Queensland University of Technology, Brisbane, QLD 4000, Australia

**Keywords:** volatile renewable resources, microbial infection, plant secondary metabolites, antimicrobial essential oils, biologically-active polymers, plasma-assisted technique

## Abstract

The persistent issue of bacterial and fungal colonization of artificial implantable materials and the decreasing efficacy of conventional systemic antibiotics used to treat implant-associated infections has led to the development of a wide range of antifouling and antibacterial strategies. This article reviews one such strategy where inherently biologically active renewable resources, i.e., plant secondary metabolites (PSMs) and their naturally occurring combinations (i.e., essential oils) are used for surface functionalization and synthesis of polymer thin films. With a distinct mode of antibacterial activity, broad spectrum of action, and diversity of available chemistries, plant secondary metabolites present an attractive alternative to conventional antibiotics. However, their conversion from liquid to solid phase without a significant loss of activity is not trivial. Using selected examples, this article shows how plasma techniques provide a sufficiently flexible and chemically reactive environment to enable the synthesis of biologically-active polymer coatings from volatile renewable resources.

## 1. Introduction

In 1963, Lieutenant W. Sanborn was the first to systematically relate surface contamination to the transmission of microorganisms [1]. Later, numerous studies have confirmed the attachment and proliferation of microbial cells on artificial surfaces, such as that of medical devices [2,3]. In spite of significant progress in the development of antibacterial and antifouling surfaces, microbial adhesion and the resulting development of a thick sessile layer, i.e., the biofilm, on the surfaces of synthetic implants remains a major issue with their clinical use [4]. Therapeutic statistics have demonstrated that approximately 80% of worldwide surgical site associated-infections may relate to microscopic biofilm formation [5]. Further, owing to microbial infection, and the subsequent failure of medical devices, there has been a significant increase in the number of revision surgeries [6,7]. In the United States alone, approximately 17 million new biofilm-related infections are reported annually, leading to approximately 550,000 fatalities each year [8].

The emergence of bacteria that are resistant to typically used antibiotics is now well recognized [9,10]. The most serious problem caused by antibiotic resistance is that some pathogenic bacteria have now become resistant to virtually all standard antibiotics [11,12]. Significant examples are methicillin-resistant *Staphylococcus aureus* (MRSA), vancomycin-resistant *Enterococcus* (VRE), multi-drug-resistant *Mycobacterium tuberculosis* (MDR-TB), and *Klebsiella pneumoniae* carbapenemase-producing bacteria [13]. Moreover, today, MRSA, a leading cause of most common hospital infections, and *Neisseria gonorrhoeae*, the pathogen responsible for gonorrhea, are almost resistant to benzyl penicillin, while in the past, these pathogens were highly susceptible to the drug [14]. The impact of microbial resistance can be diminished considerably through reduced antibiotic consumption. 

Renewable resources have attracted some research attention as precursors for developing tailored bioactive polymers that are capable of minimizing the rate of bacterial adhesion and biofilm growth in healthcare facilities. Within the therapeutic arsenal of naturally-available alternatives that have been explored, plant secondary metabolites (PSMs), such as essential oils and herb extracts, have revealed relatively powerful broad-spectrum antibacterial activities [15,16]. Good examples of currently used PSMs are tea tree (*Melaleuca alternifolia*), geranium, zataria, and cinnamon oils that have shown inherent bactericidal performance in their liquid and/or vapor form toward important pathogenic microbes. Due to the presence of a large number of active molecules within a single essential oil or plant extract, their antimicrobial pathway is not fully understood and cannot be attributed to a particular mechanism [17]. However, the pharmaceutical, cosmetic, and food industries have recently paid great attention to bioactive PSMs, by way of the usage of natural additives as a substitute for synthetic preservatives [18]. Indeed, PSMs are a relatively low-cost renewable resource available in commercial quantities, with limited toxicity, and potentially, different biocidal mechanisms to synthetic antibiotics, which make them an appropriate precursor for “green” functional polymeric materials. On the other hand, using PSMs for surface functionalization through immobilization or synthesis of coatings without loss of functionality is challenging, in part due to the issues with solubility and volatility of these precursors. The plasma-assisted technique overcomes these challenges, allowing the fabrication of a polymerized 3D matrix from renewable precursors with control over its surface properties and chemical functionality. Under appropriate fabrication conditions, plasma-enabled synthesis may help preserve/retain the inherent antimicrobial functionality of PSMs within the solid polymer-like thin films. Plasma polymers of PSMs (PP-PSMs) have several advantages including low cytotoxicity, long-term stability, and a reduced risk of developing microbial resistance. These advantageous properties render PP-PSMs a suitable candidate for bioactive coating applications.

Thus, the focus of this article is on:

- The challenge of bacterial adhesion, biofilms formation, and medical device-associated infections.
- The retention of inherent antimicrobial activity of sustainable monomers, e.g., plant secondary metabolites within solid polymers with the aim of applying them as bioactive coatings.

## 2. Microbial Contamination

Global production of medical devices and associated materials is an industry worth over $180 billion, and is expanding swiftly [19]. Microbial contamination of these biomaterials is a serious and widespread problem facing current health systems, because it often leads to devastating infections and the failure of the affected device. Adhesion of planktonic microorganisms (e.g., bacteria and fungi) to surfaces is the first stage during surface colonization, followed by the subsequent formation of biofilms which provide an ideal environment for the microbial community to flourish and effectively evade treatment. An active biofilm can be up to 1000 times more resistant to an antimicrobial treatment than planktonic bacteria of the same species [20,21]. Biofilms act as a nidus for systemic pathogenic infections, including dental cavities, periodontal disease, pneumonia associated with cystic fibrosis, otitis media, osteomyelitis, bacterial prostatitis, native valve endocarditis, meloidosis, and musculoskeletal infections [22,23]. Thus, a thorough understanding of the mechanisms by which microorganisms attach to the substrate, and the structure and dynamics of biofilm formation is necessary to develop bio-active coatings that reduce or prevent medical device-associated infections. 

### 2.1. Bacterial Adhesion

Bacterial cells are essentially capable of attaching to all natural and artificial surfaces [24]. Yet it has been assumed that bacteria favorably stick to rougher surfaces for three reasons: (i) A higher surface area available for attachment; (ii) protection from shear forces; and (iii) chemical changes that cause preferential physico-chemical interactions [25]. Also, there is consensus among scientists that the solid–liquid interface between a surface and an aqueous medium (e.g., water and blood) provides a suitable environment for the adhesion and propagation of bacteria [26].

Before the first microorganism reaches the surface, water, salt ions, or proteins that exist in the environment will adhere to the substrate because of the nature of the attachment, which is dependent on the properties of the material [27] and the chemistry of the environment. Consequently, a single layer of organic macromolecules called a ‘conditioning film’ is formed [28]. The characteristics of conditioning films in turn significantly influence the surface colonization. As the bacterial cell approaches the surface (a few nanometers), the initial stage of adhesion is governed by a number of physico-chemical effects, which include long-range and short-range forces. The long range forces include gravitational, van der Waals, and electrostatic interactions, while the short range forces include hydrogen bonding, dipole–dipole, ionic, and hydrophobic interactions [21,29]. The initial microbial attachment is considered reversible, as the cell will attach to the conditioning film not the surface itself. During adhesion to the surface, various bacteria can transiently produce flagella that render them very motile. Depending on the species, microorganisms may have appendages such as fimbriae, or polymeric fibers, also called pili or curli, which enhance attachment to surfaces [30]. For example, the curli fibres of *E. coli* are 4–6 nm wide unbranched filaments, having a distinctive morphology that can be easily detected by electron microscopy [31]. If the microorganisms are not immediately removed from the surface, they can anchor themselves more permanently by producing a large amount of fibrous glycocalyx that performs the role of ’cement‘ to attach cells to the targeted surface [32]. 

### 2.2. Biofilm Formation

**Figure 1:**
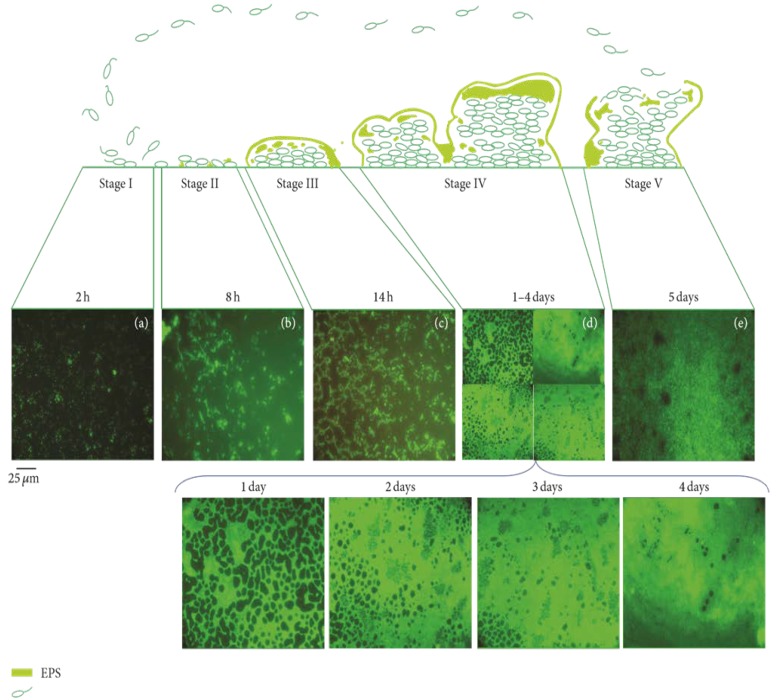
Schematic of the lifecycle of *P. aeruginosa* grown in glucose media. Images of inverted fluorescence microscopy with 400× magnification present stages of biofilm development. In stage I, planktonic bacteria attach to a solid surface. In stage II, the attachment becomes irreversible. Stage III elucidates the microcolony foundation. Stage IV illustrates the biofilm maturation and growth of the three-dimensional bacterial sanctuaries. In stage V, dispersion occurs and free planktonic cells are released from the cluster biofilm to colonize new locations. Images characterize a 250 × 250 μm^2^ field. Reproduced from [51].

After adhering to solid surfaces, the next step of permanent attachment is growing a bacterial “sanctuary”, which is the biofilm. Biofilm formation is a four stage process which includes: (i) irreversible attachment; (ii) early development; (iii) maturation; and (iv) detachment or dispersal of cells, as seen Figure 1 [29]. In the case of irreversible adhesion, major changes occur in gene/protein expression of microbial cells. It has been shown conclusively that bacteria secrete a highly hydrated layer (biofilm) that provides a shield against host defense system and antibiotics, and strengthens the attachment of the microorganisms to the surface. Early steps of biofilm formation are controlled by physical adsorption processes and evolution dynamics of planktonic pathogens [33].

A biofilm cluster consists of accumulations of extracellular polymeric substances (EPS), primarily polysaccharides, proteins, nucleic acids, and lipids [34,35]. Typically, a viable biofilm involves three organic layers. The first layer is attached to the surface of the tissue or biomaterial, the second layer is called the “biofilm base”, which holds the bacterial aggregation, and the third layer, known as the “surface film”, performs as an outer layer where planktonic organisms are released [6]. Biofilm architecture is heterogeneous both in space and time. The thickness of a biofilm varies depending on the microbial species. For example, the mean thickness of a *P. aeruginosa* biofilm is about 24 µm, while *S. epidermidis* has a mean biofilm thickness of 32.3 µm; thickness can reach more than 400 µm in some species [36]. Active biofilms are highly hydrated, with 50%–90% of the overall area at each sectioning depth comprising EPS and liquid [37]. Direct microscopic observation has shown that biofilm clusters accumulate a large quantity of pathogens within a small area, with microorganism cell densities on an infected surface reaching 10^6^ cells/cm^2^ [38]. Microorganisms communicate with each other inside a biofilm by producing chemotactic particles or pheromones, in a process called “quorum sensing” [39]. Biofilm sanctuaries can include a single infectious species or multiple infectious species, as well as non-pathogenic microorganisms which nevertheless can produce substances that would benefit the survival and proliferation of the pathogenic species. In the case of the infection of medical devices and implants, a single bacterial species is usually responsible for biofilm formation. While in environmental surfaces, groupings of various species will usually dominate the biofilm [40]. 

Hydrodynamic, physiological, and ecological conditions, along with presence of other colonizers and harmful agents (e.g., antibiotics and antimicrobial nanoparticles), considerably influence the biofilm structure. For example, biofilm structures of *P. fluorescens* and *P. aeruginosa* are significantly affected by nutritional cues, e.g., carbon and iron availability in their surroundings, respectively [41]. It has been reported that shear forces affect the distribution of microcolonies due to the passage of fluid over the biofilm. At low shear forces, the colonies are formed like a channel, while at high shear forces, the colonies are extended and susceptible to rapid vibrations [42]. These channels are essential for bacteria to transport the necessary water, nutrients, and oxygen to the bacterial community within the biofilm [43]. It has been shown that an increasing loading rate applied under a stable shear stress induced the formation of thicker and rougher biofilms [44]. Detachment is a fundamental process in biofilm development that benefits the bacterial life cycle by allowing planktonic cells to return to the environment and settle new territories [45]. Three different biofilm strategies have been suggested to elucidate biofilm detachment: (i) swarming dispersal, where planktonic cells are freed from a bacterial cluster; (ii) clumping dispersal, where aggregates of microbial cells are separated as clumps; and (iii) surface dispersal, where biofilm matrices move across infected surfaces through shear-mediated transport [46]. Detachment initiation has been proposed to initiate in response to specific endogenous or/and exogenous cues (e.g., a lack of nutrients that causes starvation, or high cell densities) [47]. The event of detachment is complex and random. In some cases, separation of large masses (cell clusters larger than 1000 μm^2^) from mature biofilms represents only 10% of the detachment process, yet accounts for more than 60% of the microorganisms detached [48]. A considerable amount of literature has been published on biofilms, yet the mechanisms of biofilm detachment are poorly understood [49,50]. Better understanding of the detachment mechanisms is necessary to accurately evaluate the spatial distribution of the bacterial cells in their environment, their ability to survive, as well as their resistance to biocides.

### 2.3. The Impact of Biofilm Formation in the Healthcare Environment

Microbial infections related to bacterial attachment and biofilm formation have been detected on various medical devices including prosthetic heart valves, orthopedic implants, intravascular catheters, artificial hearts, left ventricular assist devices, cardiac pacemakers, vascular prostheses, cerebrospinal fluid shunts, urinary catheters, ocular prostheses, contact lenses, and intrauterine contraceptive devices [52]. The three most common device-related infections are central line-associated bloodstream infection, ventilator-associated pneumonia (VAP), and Foley catheter-associated urinary tract infection (UTI) [53]. Studies have shown that 60–70% of nosocomial infections are associated with some type of an implanted medical device [54]. More specifically, the Centre for Disease Control and Prevention in the USA reported that of the infections in medical devices, 32% are urinary tract infections, 22% are surgical site infections, 15% can be attributed to pneumonia and lung infections, and 14% constitute bloodstream infections [55]. Microorganisms also form biofilms on the damaged vascular endothelium of native heart valves in patients with pre-existing cardiac disease, causing Candida infectious endocarditis [35]. It is known that biofilms of Candida species cause malfunctioning of the valve in tracheo-esophageal voice prostheses, leading to an increase in air flow resistance and potential fluid leakage [56]. Furthermore, scanning electron microscopy confirmed biofilm development at the tip of urinary catheters even after a short period of exposure [29].

While a large number of microorganisms are capable of causing infections, those that are able to survive and thrive in clean sites, such as that of clinics and hospitals, present a considerable threat [57]. These organisms include Gram-positive *Enterococcus faecalis*, *Candida albicans*, *Staphylococcus aureus*, *Staphylococcus epidermidis*, and *Streptococcus viridans*; and Gram-negative *Escherichia coli*, *Klebsiella pneumoniae*, *Proteus mirabilis*, and *Pseudomonas aeruginosa*. Prevalence of these pathogens is a serious problem in modern societies. For example, *C. albicans* causes superficial and serious systemic diseases, and is known as one of the major agents of contamination in indwelling medical devices [58,59,60]. *P. aeruginosa* is an opportunistic pathogen of immunocompromised hosts and can cause native acute and chronic lung infections that result in significant morbidity and mortality, especially in cystic fibrosis patients [61,62]. *S. aureus* and *S. epidermidis* have been shown to strongly adhere and form biofilms on metallic implants, e.g., orthopaedic screws, leading to potential device failure [6].

Overview on plant extracts:

In ancient times, plant extracts and natural oils were used in various treatment procedures as antiviral, antimitotic, and antitoxigenic agents due to their strong and broad-spectrum antimicrobial activity. These products can be extracted from all plant organs such as leaves, buds, flowers, roots, stems, seeds, fruits, bark, twigs, or wood. The earliest recorded reference to the techniques and methods used to yield essential oils is believed to be that of Ibn al-Baitar (1188–1248) [63]. Nowadays, natural oils are used in numerous pharmaceutical and therapeutic applications, including ethical medicines for colds, perfumes, make-up products, in dentistry, as food preservatives, and recently, also in the field of sustainable conservation of cultural heritage [64,65,66,67,68]. More than 250 types of these naturally generated oils are traded annually on the global market, at a value of 1.2 billion USD [69]. 

PSMs are extracted as part of highly complex mixtures of various individual constituents (often hundreds of components) [70]. PSMs were reported to contain a variety of chemical groups in their structure, such as alcohols (terpineol, menthol, geraniol, linalool, citronellol, and borneol), aldehydes (benzaldehyde, citral, cinnamaldehyde, citronellal, and vanillin), acids (benzoic, cinnamic, isovaleric, and myristic), esters (acetates, cinnamates, benzoates, and salicylates), hydrocarbons (cymene, sabinene, myrcene, and storene), ketones (carvone, camphor, pulegone, menthone, and thujone), phenol ethers (safrol and anethol), phenols (carvacrol, eugenol, and thymol), terpenes (camphene, cedrene, limonene, pinene, and phellandrene), and oxides (cineol) [71].

## 3. The Antibacterial Activities of PSMs

Even though synthetic antibiotics have been the best weapon for eradicating microbial infections since the arrival of penicillin, the overuse of these medications is gradually rendering them ineffective. It is anticipated that if new strategies are not developed soon, medical treatments could retreat to the era where slight injuries and common infections develop into a serious medical problems. One promising strategy has been inspired by the inherent bioactivity of plant secondary metabolites [72]. It is known that most plants produce these organic molecules as antimicrobial agents to combat harmful microorganisms [73,74]. In the past few decades, the progress in the synthesis of nanoscale materials, in particular plasma-assisted fabrication provides the means to retain the antimicrobial activities of PSMs within bioactive coatings. This family of techniques is compatible with PSMs, and offers several advantages such as being an environmentally friendly, versatile, and low-cost technology (discussed further in this article).

In their liquid form, lavender, garlic, oregano, lemongrass, and cinnamon oils are good examples of naturally-occurring substances with strong antibacterial activity [75,76]. Their individual constituents, e.g., citronellol and geraniol are aromatic acyclic monoterpene alcohols that are very powerful bactericides [77,78,79,80]. Terpinene-4-ol, a major component of tea tree oil, is a broad-spectrum nonspecific biocide well-known as a natural agent against microbial species such as *E. coli*, *P. aeruginosa*, *Acinetobacter baumannii*, and several drug-resistant bacteria (e.g., MRSA) [81]. A number of PSMs have been used against cancer cells, whereas others are currently used in food preservation [82,83]. In their vapor phase, a number of PSMs have demonstrated strong antibacterial activities [84,85]. So far, there are thousands of natural oils currently known. Among them, 300 oils are important and commonly used in the pharmaceutical, food, sanitary, agronomic, perfume, and cosmetic productions [86].

### 3.1. The Antibacterial Mechanisms of PSMs

**Figure 2:**
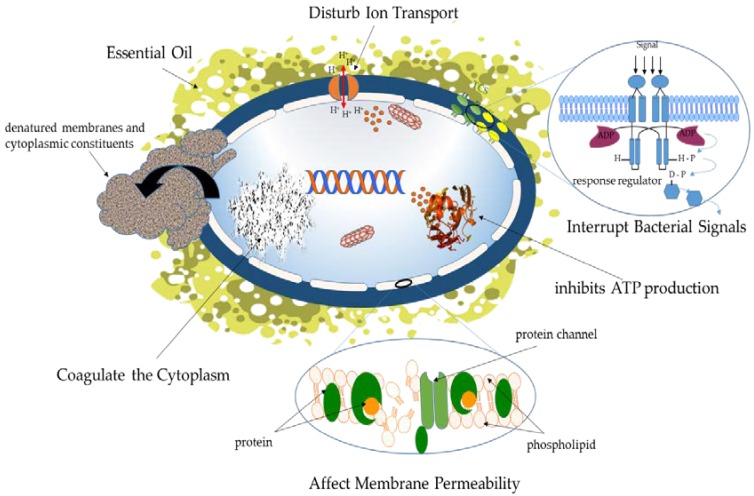
Scheme represents the proposed antibacterial mechanisms of secondary planet metabolizes in their liquid form.

The antibacterial action of PSMs (in their liquid form) is complex and not yet fully understood; it potentially involves several mechanisms, as summarized in Figure 2. A number of researchers have proposed that the hydrophobic nature of PSMs allows them to accumulate and perturb the structure and function of lipids of the microbial cell membrane, disturbing biological function, and causing the failure of chemiosmotic control, thus rendering the membrane more permeable [75,87]. An increase in membrane fluidity and permeability results in membrane expansion and the damage of membrane-embedded proteins which triggers inhibition of the respiration system and alteration of ion transport activities of bacterial cells [88]. For example, carvacrol oil was reported to make the cell membrane permeable to K^+^ and H^+^, consequently dissipating the proton motive force and inhibiting ATP production [89]. Similarly, menthol and citronellol cause an expansion of the cell membrane, leading to the passive diffusion of ions between the stretched phospholipids [69]. Ultee and Smid (2001) hypothesized that during exposure to PSMs, the driving force for the optimal secretion of the toxin (ATP or the proton motive force) is not sufficient, causing accumulation of the toxin inside the cell, which in turn inhibits normal microbial metabolism [90]. Some active PSMs are capable to coagulate the microbial cytoplasm, leading to cell inactivation [91]. For example, it has been observed that coagulated materials (related to denatured membranes, cytoplasmic constituents, and proteins) were formed outside of the bacterial body when cells (*E. coli*) were grown in the presence of tea tree oil. These coagulates were released through microscopic holes produced in the cell wall as a result of the interaction with the oil [92].

Exposure to PSMs can lead to the reduction in enzymatic activities, loss of turgor pressure, changes in DNA synthesis and inhibition of different metabolic functions [89]. Moreover, some oils, such as rose, geranium, lavender, and rosemary have been shown to inhibit cell–cell communication, affecting the quorum sensing (QS) network in the bacterial community [93]. The QS system is vital for bacterial growth, and hence, any interference with the sensing network may reduce pathogenicity, biofilm formation, and antibiotic resistance during infection events. 

The antimicrobial performance of PSMs is linked to their chemical structure, particularly the presence of –OH functional groups [94]. Each compound may reveal a different biocidal mechanism toward microorganisms [69]. The bioactivity of several active oils is associated with the presence of phenolic groups. For example, the antimicrobial efficacy of clove, thyme, and oregano oils is related to the presence of phenol-containing eugenol, thymol, and carvacrol, respectively [95]. However, other findings indicate that the components present in high quantities in the oil are not necessarily responsible for the entire biological activity of a PSM. The antibacterial performance of these complex mixtures relies on a variety of synergistic effects of different sub-components in the oil. Furthermore, it can also be attributed to the presence of other components that may be effective even in small quantities [96,97]. In the case of essential oils containing a high percentage of phenolic compounds (e.g., carvacrol, thymol), it can be assumed that their bactericidal action would be similar to other phenolic groups, e.g., by way of the disturbance of the membrane, disorder the proton motive force, electron flow, and coagulation of cell contents [87]. In the case of complex mixtures, where numerous active molecules are present, potential synergistic and antagonistic influences, as well as minor compounds that can have an important contribution to the oil’s activity, need to be considered [64,98]. It is important to state that the biocidal mechanisms of PSMs are dissimilar from currently used synthetic antibiotics, which should minimize the likelihood of the development of microbial cross-drug resistance [99].

### 3.2. Sustainable Polymers from Bioactive Essential Oils

The ecological concerns of current petroleum processing, along with the economic recession, depleting reserves, and political aspects, have led to increased interest in the production of sustainable polymers derived from renewable resources [100,101]. These eco-friendly polymers can be derived from a wide range of possible precursor materials, including oxygen-rich monomers (e.g., carboxylic acids), hydrocarbon-rich monomers (fatty acids, terpenes, and vegetable oils), and non-hydrocarbon monomers (carbon dioxide) [102]. So far, polymers derived from essential oils, vegetable oils, bio-ethanol, cellulose, fats, resins, naturally occurring polysaccharides, microbial syntheses, and other natural ingredients have been widely used for a variety of applications [103,104,105,106,107,108]. Essential oils, in particular, are renewable in nature, relatively inexpensive, available in commercial quantities, and display minimal toxicity compared to many conventionally-used precursors, which make them an appropriate precursor for “green” functional materials. Among them, terpenes (major components in a large number of essential oils) have received considerable attention. Their structure contains one or more carbon–carbon double bonds, showing a carbon skeleton of isoprene. The abundance of double bonds allows for cationic and radical polymerization of terpenes, along with epoxidation as a path to biodegradable oxygenated polymers [109]. Cationic polymerization has been generally accepted to be the most appropriate kind of chain reaction for these monomers [110]. However, essential oils have not been widely applied to the production of bioactive polymers due to limitations associated with fabrication systems and oil properties [111,112]. These limitations include challenges in controlling the surface chemistry and morphology of the synthesized materials, and solubility and/or volatility of the natural monomers.

Recent technological advances in the field of controlled polymerization, catalysis, nanoencapsulation, and effective organic functionalization, give great potential for the application of essential oils in manufacturing of sustainable polymers with innovative designs and characteristics. This allows the fabrication of organic films with good control over film thickness, physico-chemical properties, and, importantly, biological functionality. For instance, it was possible to successfully engineer antibacterial UV-cured networks by using a thiol-ene route with covalent immobilization of natural terpenes (linalool and a trithiol) as antibacterial agents, without employing any organic solvent. These bio-based materials exhibited attractive thermal properties, were not affected by water penetration under high moisture conditions, and displayed strong inhibition of microorganisms [113]. Chen et al., (2012) developed the reversible transfer polymerization approach to design a series of cationic rosin-containing methacrylate bioactive-copolymers. The antibacterial activities of these rosin-containing copolymers were found to be dependent on both the degree of quaternization of the rosin group, the molecular weight of copolymers, and the conformation of hydrophobic group [114]. Furthermore, a cinnamon essential oil/cyclodextrin integrated into a polylactic acid nanofilm made by electrospinning and co-precipitation showed strong antimicrobial activity [115].

Several studies have been carried out in order to incorporate active essential oils into selected polymers through applying emulsification or homogenization methods, where ultra-fine emulsions of oils are formed containing polymer at the continuous aqueous phase. Upon drying, lipid droplets remain incorporated into the polymer structure. The releasing rate of the embedded-oils from films is subject to multiple factors, such as electrostatic interactions between the oil and the polymer chains, osmosis, structural variations induced by the presence of the oil, as well as environmental circumstances [116]. Remarkably, a small fraction of an incorporated essential oil within a polymer structure is sufficient to achieve the desired antimicrobial properties. For example, quince seed mucilage films containing a low percentage (1.5–2%) of oregano essential oil were reported to be very effective against several microorganisms, including *S. aureus*, *E. coli*, and *S. putrefaciens* [117]. Other findings showed that inactive chitosan films were transformed into bioactive materials when a small quantity (~1–2%) of extract from two endemic herbs (*Thymus moroderi* or *Thymus piperella*) were integrated within the films [118]. 

Encapsulation of oils has been developed as one such technology that has great potential to improve the physical stability of the active components, protecting them from degradation due to environmental aspects (e.g., oxygen, light, moisture, and pH) [119]. Among the nanometric encapsulation structures currently being used, nanoemulsions are mainly utilized due to the possibility of formulation with natural components and the compatibility with industrially scalable manufacturing processes by high pressure homogenization [120]. Nanoemulsions are defined as emulsions with ultra-small droplet sizes of approximately 100 nm. At this scale, there is a potential of enhancing physico-chemical properties and stability of the active compound. In addition, the oil bioactivity can be considerably increased, since significant increases in the surface area per unit of mass can be achieved, improving the passive mechanisms of cell absorption, which again allows for the reduction of the oil quantity required to ensure antimicrobial action [121]. The encapsulated essential oils are promising antimicrobial agents for biodegradable/edible coatings in food packaging industries to inhibit pathogenic microorganisms [122]. It has been reported that the encapsulation in nanoemulsion formulation of a terpenes mixture and limonene increased the antimicrobial performance of the pure compounds against various microorganisms such as *E. coli* and *Saccharomyces cerevisiae*, through increases of transport mechanisms in the membrane of the target cell [120]. Mohammadi et al., (2015) also encapsulated *Zataria multiflora* essential oil in chitosan nanoparticles (average size of 125–175 nm) and reported a controlled and sustained release of essential oil for 40 days, along with a superior antifungal performance in comparison with the un-encapsulated oil [123]. Moreover, films with 1.5% nanocomposite marjoram oil diminished the numbers of *E. coli*, *S. aureus*, and *Listeria monocytogenes* populations with respect to the control of up to 4.52, 5.80, and 6.33 log, respectively [124]. Similarly, introduction of carvacrol nanoemulsions into modified chitosan have led to the development of a bioactive film, which was active against Gram-negative pathogenic bacteria [125]. 

It is worth mentioning that, in many cases, the vapor phase of essential oils exhibits strong inhibitive performance against pathogens, even more effective than direct application [126,127]. For instance, Avila-Sosa et al. (2012) found that chitosan films incorporating cinnamon or Mexican oregano essential oils can inhibit fungi by vapor contact at lower oil concentrations than those required for amaranth and starch polymeric coatings [116].

### 3.3. Plasma-Assisted Fabrication of PSMs

Among fabrication techniques, cold plasma polymerization is a multipurpose approach that is a relatively fast and low-cost method for fabricating coatings from a wide array of natural precursors, including those that do not usually polymerize by conventional methods, and do not require further chemical or physical processing (e.g., annealing and catalysts) [128]. From a processing point of view, many PSMs are compatible with plasma polymerization, which is in essence a chemical vapor deposition process enhanced by the catalytic activity of plasma, because they are highly volatile at room temperature no external heat or carrier gas are required to deliver the precursor macromolecules to the fabrication zone. 

Introduction of PSMs molecules, in vapor phase, into a highly reactive plasma field triggers a wide range of reactions including fragmentation, oligomerization, rearrangement, and polymerization. The degree of dissociation is highly dependent on the amount of energy provided into the plasma system and the pressure in the chamber. Fragmentation is initiated by active electrons rather than thermal excitation or chemical reactions, creating a unique mixture of chemically diverse species (e.g., unsaturated bonds, ions, neutrals, and free radicals), which may not be reachable under other conditions [129]. It is believed that weakly ionized plasma and relatively low substrate temperature during deposition promotes condensation and adsorption of non-excited species, which help to increase the proportion of non/partially-fragmented precursor molecules on the substrate [130]. The recombination of the reactive species and precursor molecules may lead to the formation of the organic thin layer (polymer) on the surface of a given substrate. Due to the diversity of functional groups and reactive species, the polymer can be formed in several ways, involving free-radicals and induced-polymerization of fragments containing unsaturated carbon–carbon bonds; recombination fragment/recombination is initiated by the plasma-generated and surface-attached reactive ions [131]. The formed polymer is often highly branched and highly cross-linked (amorphous), comprising large quantities of trapped free radicals in its structure [132]. 

**Figure 3:**
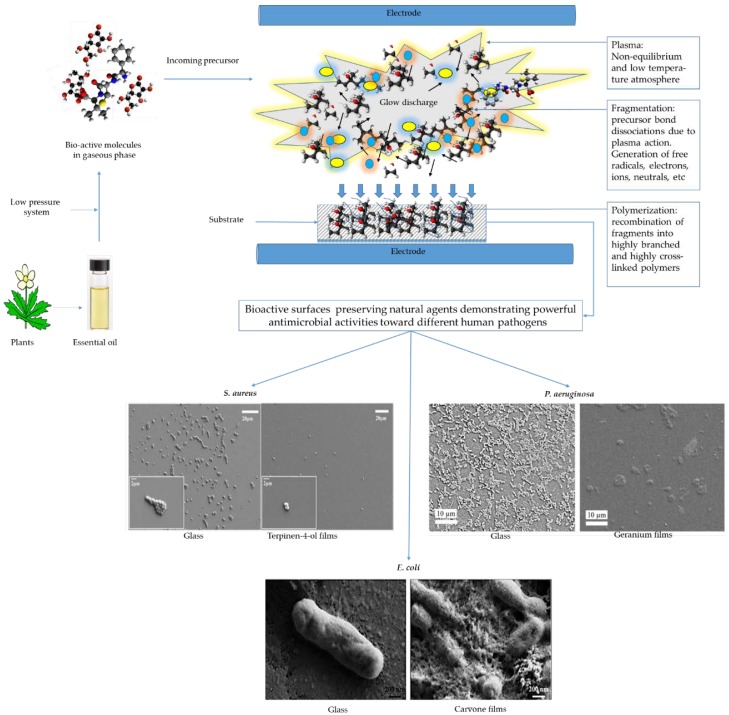
Representative examples of plasma polymerization of plant secondary metabolites, where retention of the antimicrobial activity was achieved. As soon as a bioactive secondary plant metabolite (or an essential oil) is placed under low pressure, the molecules gain sufficient kinetic energy to separate and begin independently moving towards the glow region within the deposition chamber. Exposure of the molecules to the highly reactive plasma initiates various chemical reactions, such as bonds fragmentation, oligomerization, and polymerization. At the chosen plasma parameters, the process allows for the preservation of active functional groups of PSMs within the cross-linked solid polymeric films. Direct observations of SEM demonstrated the powerful antimicrobial performance of geranium, terpenen-4-ol, and carvone films in contact with different pathogens. The antimicrobial activities of these films included antibiofouling effects and/or bactericidal actions (e.g., membrane distortion, pores creation, and membrane damage). The SEM images are reproduced with permission from [137,144,153].

A large number of species that exists in the discharge (e.g., ions, electrons, stable molecules, radicals, and photons) can react with each other and the forming chains through a range of interaction mechanisms, as seen in Figure 3. The complexity of the process of PSM plasma makes the evaluation of each specific reaction, along with the prediction of material properties, very challenging. In some cases, few specific reactions can dominate the formation of the film, especially at low input power. Thus, it is rational to propose that films fabricated from PSMs, using plasma under specific deposition conditions (e.g., specific input power, frequency, flow rate, and temperature), could retain some/most of the functional groups of the original PSMs within the bioactive three-dimensional solid film. In addition, the unfragmented precursor molecules trapped within the polymer during the fabrication may elute over time, acting as a drug release coating, with the capacity to retard microbial attachment and biofilm development on the surface [133]. 

A number of attempts have been made to manufacture antibacterial surfaces, based on plasma polymerization of essential oils, where antibacterial performance is based only on the natural bioactivity of the polymerized surfaces, in the absence of synthetic additives, inorganic nanoparticles, or conventional antibiotics. Using this information, we strongly encourage the reader to further research this rapidly growing and highly-promising arena. Here, we highlight the successful manufacturing of antimicrobial coatings from different PSMs using the cold plasma polymerization technique.

#### 3.3.1. Terpinen-4-ol

Terpinen-4-ol is a monocyclic terpene alcohol that is an active component of tea tree oil. Terpinen-4-ol has demonstrated powerful antimicrobial and anti-inflammatory properties [134,135]. Upon interaction with microorganisms, cyclic terpene hydrocarbons have been shown to accumulate in the cell membrane. This disturbs membrane integrity, triggering an increased passive flux of protons through the membrane and dissipation of the proton motive force [136]. Bazaka et al. (2011) prepared plasma polymerized coatings derived from terpinen-4-ol at various input power levels, showing a considerable potential in minimizing bacterial attachment and metabolic activity of *S. aureus* and *P. aeruginosa*. Fabrication at a low input power level, 10 W, resulted in a partial retention of biologically-active groups of the original precursor, which led to significant antimicrobial and antibiofouling activities of the terpenol-derived coatings [137]. Confocal laser scanning microscopy evidently showed that around 90% of *S. aureus* cells retained on the films of the 10 W substrata were non-viable, in comparison to that retained on the surface of 25 W films [138,139]. However, when fabricated at higher input power (25 W), these films lost their biocidal activity, and promoted adhesion and proliferation of tested bacterial cells and biofilm development. In a recent report, the decrease in antibacterial activity with increasing radio frequency (RF) energy was also observed in the plasma polymerization of polyterpenol films [140].

#### 3.3.2. Carvone

Carvone is found in various essential oils, such as caraway, spearmint, and dill. This PSM has shown a variety of antiproliferative effects with regards to microbial cells; likely due to the presence of a monoterpene group in its structure [141,142]. In addition, carvone and its related compounds were shown to be potential chemopreventive agents, due to their ability to induce increased activity of detoxifying enzymes. The α,β-unsaturated ketone system in carvone is generally expected to be responsible for the high enzyme-inducing action [143]. Recently, Chan et al., (2016) fabricated polymer coatings resultant from plasma polymerization of carvone [144]. At an input power of 10 W, carvone polymerized coatings demonstrated almost equal antimicrobial performance against both Gram-negative and Gram-positive bacteria (86% decrease in *E. coli* and 84% reduction in *S. aureus*), with no cytotoxic effect towards primary human endothelial cells. In addition, these coatings were smooth, highly cross-linked hydrocarbons, with low fractions of carboxyl, hydroxyl, and amine-amide functionalities. Although the carvone surfaces reduce bacterial adhesion, it was observed that some cells were damaged and died after attaching to the surface. The scanning electron microscope (SEM) images clearly exhibited membrane distortion, pore creation, and membrane rupture of microorganisms attached on the surface of plasma polymers of carvone.

#### 3.3.3. Eucalyptol

Eucalyptol, a major component of eucalyptus oil and a minor component of tea tree oil, is a saturated monoterpene known by a variety of synonyms, such as 1,8-cineole, 1,8-epoxy-p-menthane, and cajeputol. This PSM has been demonstrated to retain strong biological activities, including anti-inflammatory, antifungal, antibiofilm, and antiseptic properties toward a range of bacteria [145,146,147,148]. The retention of the natural bio-active groups of the 1,8-cineole oil was also achieved using plasma polymerization. Fabricated at 20 W, moderate hydrophobic coatings were achieved, with the ability to reduce the attachment of *E. coli* and *S. aureus* cells by 98% and 64%, respectively, compared to unmodified glass. In addition, the 1,8-cineole plasma films resisted biofilm formation after 5 days of incubation in the presence of bacterial cells. The polymer surface and any products that may be released from the film were also found not to be cytotoxic to mammalian cells [149]. In the same way, Mann and Fisher (2017) used a range of applied RF powers (P = 50–150 W) and H_2_O_(v)_ plasma-treatment during the plasma fabrication of 1,8-cineole polymers. The fabricated films retained some antimicrobial behaviors characteristic of the precursor, in addition to the desired properties, such as being highly adherent to the substrate, conformal, and with smooth surfaces. The in vitro studies showed that *E. coli* were largely nonviable and unable to colonize the plasma–cineole surface over the 5 day biofilm development assay period. The biofilm coverage on these surfaces was significantly lower (<10%) than the glass control [150].

#### 3.3.4. Geranium

Geranium (*Pelargonium graveolens*) oil produces a mixture of various components (more than 80), such as linalool, citronellol, and geraniol [151]. Studies have revealed that geranium oil is able to combat pathogens, including both Gram-negative and Gram-positive bacterial strains [82,152]. More recently, geranium oil-derived coatings were also found to have the potential to reduce the microbial adhesion and biofilm formation of select human pathogens, such as *S. aureus*, *P. aeruginosa*, and *E. coli*. The input RF power, in particular, played a substantial role in controlling the surface biochemistry and extensively enhanced the biocidal activity of the fabricated coatings. Films deposited at 10 W caused a significant decrease in the number of cells, biovolume, and biofilm thickness. In contrast, there was no significant change in the bacterial colonization between films fabricated at 50 W and an unmodified glass control. In addition to their biological activities, geranium polymer films showed several advantages, including low density, uniform coverage, good adhesion, and considerable physical stability [153,154].

Despite the fact that the mechanism by which the deactivation process takes place is not fully understood, the attractive antibacterial performance of PP-PSMs surfaces indicate that the original active chemistry of the oils are partially retained within the structure of the fabricated films. Undeniably, plasma parameters are the key factors that determine the extent of retention of biological functionality. The degree of precursor fragmentation is directly related to the amount of applied energy (RF power). For example, during the polymerization of geranium oil and terpinen-4-ol, a slight increase in the input power resulted in the failure to preserve the desired functional groups within the polymer. One reason for this loss of bactericidal activity could be the complete dissociation of the precursor functionalities upon plasma exposure. Furthermore, these polymerized films demonstrated a wide range of functional groups in their structure, such as primarily methyl/methylene functionalities, as well as hydroxyl, alkene, and carbonyl groups. The hydroxyl group particularly is broadly accepted to be an antimicrobial agent of polymer surfaces. It was previously reported that *S. aureus* cells do not preferentially attach to polymers comprising –OH functionality than those bearing carboxylic and methyl groups [155]. However, other surface parameters should be carefully considered during plasma fabrication. It is well known that surface chemistry, hydrophobicity, free energy, and architecture of polymer films have the potential to significantly influence the final antibacterial outcome. The synergistic effects of these parameters may determine the inhibition of bacterial attachment and proliferation.

### 3.4. Properties of PSM-Derived Polymers

For a successful polymeric antibacterial coating to satisfy the requirements of biomedical applications, the material should possess a range of specific biological, physical, and chemical properties. Films fabricated from PSMs display a wide range of desired properties, including optical transparency, moderate hydrophilicity, relatively high degradation temperature, low post-annealing retention, and good biocompatibility, forming simple, useful, and versatile bioactive coatings. Hence, a brief description of some important physico-chemical characteristics of PP-PSMs fabricated at a low input power (below 100 W) is provided below.

As a general trend observed in PP-PSMs, polymers deposited at a higher input power are typically less susceptible to mechanical deformation. This trend is owing to an increase in the degree of cross-linking correlated with higher input power, and hence, films are likely to be more stable and less susceptible to wear [156]. Highly cross-linked polymers are expressively more stiff and dense compared to conventional polymers (amorphous or crystalline arrangements). This is related to the vibrational movement of the carbon backbone of the polymeric structure that is constrained by the presence of a multiple covalent bonds between polymer chains [157].

The topographical features of PP-PSMs fabricated at suitable parameters have been shown to be uniform, pinhole free, with films being highly-adherent to the substrate [154,158,159,160,161,162,163]. The uniformity indicates that polymerization reactions occurred on the surface of the substrate in preference to the gas phase. Moreover, ultra-smooth surfaces (with an average roughness of less than 1 nm) were attained for plasma polymerization of various PSMs, which is a particularly significant factor that may influence the initial microbial adhesion [164,165]. It is worth to mention that the properties of the surface of plasma polymerized films make them highly susceptible to growth conditions, especially the input power, where more energetic ions can cause more surface bombardment and etching. Furthermore, the chosen precursor plays an important role in determining the overall surface properties, since to a degree, it defines the chemical functionalities and determines the quantities of free radicals in the plasma system [166,167]. 

A large number of plasma polymers developed from PSMs were reported to have favorable optical properties. Although optical properties were affected by processing parameters during film deposition, PP-PSMs were found to be optically transparent in the visible region and have high absorption in the infrared region. The refractive index and extinction coefficient were in the range of 1.5 and 0.001 (at 500 nm), respectively [168,169]. In addition, PSMs-derived polymer materials had optical energy gap (*E_g_*) values in the insulating and semiconducting region. For example, films fabricated from terpinen-4-ol, linalool, γ-terpinene, and geranium have *E_g_* = 2.5, 2.9, 3.0, and 3.6 eV, respectively [153,165,170]. It is important to note that the optical properties of plasma films are characteristically dependent on the structure of the *p*-conjugated chains in both the ground and excited stats, as well as on the inter-chain orientation [171]. 

In general, PP-PSMs were moderately hydrophilic, with values of contact angles ranging from ~50° to 80°. The wetting characteristics were defined largely by plasma conditions and the chemistry of the chosen precursor. For example, improved hydrophobicity of the surface was observed for films fabricated from γ-terpinene when increasing the deposition RF power from 61.0° (10 W) to 80.7° (75 W). This polymer showed strong electron donor and negligible electron acceptor behavior [172]. The range of contact angle values of the plasma polymer are well-suited for biological uses, since they enable and promote the adhesion of various cell types [153].

Given their potential application as an antibacterial coating for implants, the cytocompatibility of PP-PSMs was examined for several types of mammalian cells. A study that tested the biocompatibility of coatings fabricated from various oils (e.g., limonene, tea tree, lavender, and eucalyptus) at different deposition powers showed minimum toxic effects. After being implanted in mice for 3 days, 14 days, and 28 days, all PP-PSM films were demonstrated to be biocompatible. While in most cases, these coatings did not produce an unwanted host or material response, in a number of cases mice sinus formation was observed, however, it was deemed not significant [173]. The biocompatibility of polymer films is a property that should be addressed carefully for protective coatings in medical applications, in particular for implantable devices, since the film surface directly interfaces with various bio-components including blood, proteins, cells, and tissue growth. Hence, non-biocompatible coatings may lead to failure, toxic responses, abnormal cell/tissue responses, and device degradation. 

It should be noted that PP-PSMs have shown some limitations. For example, they are generally insoluble in organic solvents owing to their high degree of cross-linking. This feature, in particular, greatly complicates the characterization of the polymers [131]. It was also observed that PP-PSMs are highly susceptible to changes brought by the chemical composition of the medium (e.g., the aqueous solution and body fluid) that may affect their operation in some applications [174].

Essential oils variations:

Generally, it is accepted that the chemical composition of essential oils varies by plant health, growth stage, climate, edaphic factors, and harvest time. On the other hand, the degradation kinetics of these oils, due to external factors (e.g., temperature, light, and atmospheric oxygen exposure, presence of impurities), should be thoroughly taken into account [175]. For example, pure cinnamaldehyde was reported to decompose to benzaldehyde at temperatures approaching 60 °C. But, once it combined with eugenol or cinnamon leaf oil, cinnamaldehyde remained stable at 200 °C [176]. The molecular structures of natural oils have a substantial effect on the degree of degradation. Compounds rich in allylic hydrogen atoms could be potential targets for autoxidation, where hydrogen atom abstraction is giving rise to resonance-stabilized radicals, which are highly preferable due to their lower activation energy [177]. Furthermore, essential oil components are generally known to easily convert into each other (through processes such as isomerization, oxidation, cyclization, or dehydrogenation reactions), because of their structural relationship within the same chemical group [175,177].

It is important to mention that several essential oils (e.g., tea tree, lavender, and terpenene-4-ol) have shown some irritation and allergies in users (via inhalation or direct contact) [178,179,180]. The allergic reactions typically arise from certain components such as benzyl alcohol, cinnamyl alcohol, iso-eugenol, eugenol, hydroxycitronellal, geraniol, and various others constituents [181,182,183]. However, sensitive symptoms due to essential oils can range from relatively minor incidences of irritation and sensitization, to contact dermatitis and the most serious anaphylactic reaction, thus, should be well considered [184,185].

## 4. Challenges

In the scientific and manufacturing field, replication or reproduction of consistent systematic results is the key to success [186]. A major issue of plasma techniques is the constancy of the result, particularly across different plasma systems, due to differences in processing parameters (e.g., power, pressure, temperature, flow rate, and tube geometry). For example, changes in the design of plasma equipment can affect the flow dynamics of vapors through the system, and the profile of the plasma discharge zone, which could potentially alter the nature, homogeneity, and density of the gas phase species inside the reactor. Indeed, this problem becomes more obvious during the fabrication of functional coatings from PSMs, where retention of certain chemical moieties is essential [187]. To minimize the variation of films produced across different plasma systems, a scaling factor route can be applied that takes into account both the actual energy consumed in the active plasma field, and the differences in the geometry of the utilized reactors [188]. 

Another concern comes from the varying properties of the renewable precursor. It is well documented that essential oil composition is very complex and depends on multiple interacting factors. In addition, De Masi et al. (2006) reported that the chemical compounds of essential oils were found to be extremely variable in various cultivars/genotypes of the same plant species and they these differences were not necessarily correlated with genetic relationships [189]. Additionally, the oil quality and biological activity can be affected storage conditions, e.g., temperature [190]. The potential to obtain biopolymer films with consistent properties regardless of the base material source, method, or time of harvest is important for successful integration into industrialized processes. 

As mentioned previously, typical plasma polymerization of PSMs (continuous mode) yields the fragmentation of large quantities of precursor molecules. The random recombination of fragments, radicals, and atoms renders the chemical structure and configuration completely irregular. In fact, the density of desired functional groups remain relatively insufficient even if the used fabrication power is low. Pulsed-plasma polymerization can address this issue. This technique offers a sequence of on-periods (a few μs-long periods during which fragmentation takes place) and off-periods (μs to ms-long periods during which recombination and polymerization occurs), where the resultant polymer should consist of more chemically regular structures than those of the continuous mode [191]. The idea is to further reduce the degree of dissociation/fragmentation of the precursor molecules, and hence the off-period reactions contributing more non-fragmented functionalities into the formed polymer. To date, the pulsed-plasma polymerization has not been used for the synthesis of antibacterial surfaces from PSMs. We highly encourage researchers to explore and expand the usage of the pulsed-plasma method, where the optimization of the desired functionality will essentially include the increasing/decreasing the off-period in pulsed polymerization. 

## 5. Conclusions

A better understanding of the way to preserve/retain the bioactivity of essential oils within a thin film is critical for the development of a wide range of bactericidal coatings suitable for medical devices. The aforementioned polymer materials that were derived from renewable resources present a promising approach toward producing antimicrobial and biocompatible materials and tissue contact coatings. However, information on the long-term performance of plasma polymerized PSMs thin films requires further exploration. Also, although a small number of systematic studies showed promising antimicrobial activity using encapsulating essential oils, further research in this direction is warranted.
